# Norepinephrine inhibits cell cycle re‐entry of neonatal rat ventricular cardiomyocytes characterized by the absence of de novo nestin expression

**DOI:** 10.14814/phy2.70488

**Published:** 2025-07-30

**Authors:** Adrien Aubry, Mariana Kebbe, Aya Al‐Katat, Louis Villeneuve, Angelino Calderone

**Affiliations:** ^1^ Montreal Heart Institute Université de Montréal Montreal Quebec Canada; ^2^ Département de Pharmacologie et Physiologie Université de Montréal Montreal Quebec Canada

**Keywords:** cell cycle re‐entry, neonatal rat ventricular cardiomyocytes, nestin, p38α/β MAPK, PKC isoforms, β_1/2_‐adrenergic receptors, mM

## Abstract

The p38α/β MAPK inhibitor SB203580 in the presence of a phorbol ester (protein kinase C activator) induced cell cycle re‐entry of two subpopulations of neonatal rat ventricular cardiomyocytes (NNVMs) distinguished by the absence or de novo nestin expression. Recent studies have reported that the β‐adrenergic receptor inhibited the cell cycle re‐entry of ventricular cardiomyocytes. The present study tested the hypothesis that sympathetic recruitment of p38α/β MAPK signaling suppressed cell cycle re‐entry of one or both subpopulations of 1‐day‐old NNVMs. Norepinephrine (NE; 1 μM) or Isoproterenol (ISO; 1 μM) treatment of NNVMs for 24 h significantly attenuated 5‐bromo‐2′‐deoxyuridine incorporation, and NE increased p38α MAPK phosphorylation. SB203580 (10 μM) treatment of NE/ISO‐stimulated NNVMs selectively re‐established the cell cycle re‐entry of the subpopulation distinguished by the absence of de novo nestin expression. Phorbol 12,13‐dibutyrate (PDBu; 100 nM) treatment attenuated 5‐bromo‐2′‐deoxyuridine incorporation, and SB203580 treatment predominantly initiated re‐entry into the cell cycle of the NNVM subpopulation characterized by de novo nestin expression. siRNA‐mediated PKC‐α protein downregulation in NNVMs selectively suppressed PDBu/SB203580‐mediated de novo nestin and subsequent cell cycle re‐entry. Thus, sympathetic stimuli acting via the β_1/2_‐adrenergic receptor selectively inhibited the cell cycle re‐entry of the nestin^(−)^‐NNVM subpopulation via recruitment of p38α/β MAPK.

## 1 INTRODUCTION

The ischemically damaged adult mammalian heart is unable to mount a cardiac regenerative response as the preponderance of ventricular cardiomyocytes exhibit a polyploidy phenotype preventing cell cycle re‐entry (Gan et al., 2020). Despite the latter paradigm, work from our lab identified a paucity of ventricular cardiomyocytes residing at the peri‐infarct region of the ischemically damaged adult rodent and human heart that were predominantly mononucleated and exhibited a unique cellular feature characterized by the appearance of the intermediate filament protein nestin (El‐Helou et al., 2008; Hertig et al., 2019; Kebbe et al., 2023). By contrast, nestin was not detected in ventricular cardiomyocytes identified in the normal adult rodent heart (El‐Helou et al., 2008; Hertig et al., 2019; Kebbe et al., 2023). Collectively, the latter observations provided the impetus to assess the underlying events implicated in the expression of the intermediate filament protein and further elucidate the potential physiological role of nestin‐expressing mononucleated ventricular cardiomyocytes in the ischemically damaged adult mammalian heart. Work from our lab revealed that the 72‐h treatment of mononucleated neonatal rat ventricular cardiomyocytes (NNVMs) with phorbol 12,13‐dibutyrate (PDBu) in the presence of the p38α/β MAPK inhibitor SB203580 led to de novo nestin expression and re‐entry into the S‐phase and G_2_‐M phase of the cell cycle (Hertig et al., 2018; Kebbe et al., 2023; Meus et al., 2017). Unexpectedly, the latter pharmacological approach also promoted the cell cycle re‐entry of a minor subpopulation of NNVMs distinguished by the absence of nestin expression (Kebbe et al., 2023). To assess the biological role of nestin, AAV9‐shRNA‐mediated inhibition of de novo expression of the intermediate filament protein significantly attenuated PDBu/SB203580‐mediated cell cycle re‐entry of NNVMs (Hertig et al., 2018). Consistent with the latter paradigm, lentivirus‐shRNA mediated downregulation of constitutively expressed nestin in rat embryonic ventricular cardiomyocytes (H9c2 cells) suppressed cell cycle re‐entry (Meus et al., 2017). Nestin expression was also identified in a subpopulation of proliferating mononucleated embryonic mouse ventricular cardiomyocytes during cardiogenesis (Hertig et al., 2018; Meus et al., 2017). De novo nestin synthesis in NNVMs also led to the acquisition of a migratory phenotype and the intermediate filament protein was detected in migrating mouse mononucleated embryonic cardiomyocytes during nascent trabeculae formation (Bergeron et al., 2021; Hertig et al., 2018; Meus et al., 2017). The cell cycle re‐entry of nestin^(−)^‐ and nestin^(+)^‐NNVMs was recapitulated in vivo following ventricular apex resection of the 1‐day‐old neonatal rat heart (Hertig et al., 2019). Moreover, SB203580 administration to the apex‐resected 1‐day‐old neonatal rat heart led to the partial replacement of the fibrin clot with newly generated myocardium characterized by a significant increase in the density of nestin^(+)^‐NNVMs and further potentiated cell cycle re‐entry of both NNVM subpopulations (Hertig et al., 2019). Lastly, the antiproliferative role of p38α/β MAPK was not limited to the neonatal rat heart as the serine/threonine kinase attenuated the cell cycle re‐entry of neonatal/adult mouse ventricular cardiomyocytes and adult zebrafish ventricular cardiomyocytes in vitro and in vivo (Engel et al., 2005, 2006; Jopling et al., 2012). Thus, the aforementioned data support the premise that p38α/β MAPK signaling in the ischemically damaged adult mammalian heart may concomitantly suppress the cell cycle re‐entry of both nestin^(−)^‐ and nestin^(+)^‐ventricular cardiomyocyte subpopulations.

The sympathetic system acting preferentially via the β‐adrenergic receptor inhibited the proliferative response of embryonic and juvenile mouse ventricular cardiomyocytes (Feridooni et al., 2017; Liu et al., 2019; Sakabe et al., 2022). The in vivo administration of the non‐selective β_1_/β_2_‐blocker propranolol reduced the percentage of binucleated mouse ventricular cardiomyocytes, thereby conferring greater cardiomyocyte endowment (Liu et al., 2019). The latter response was attributed in part to propranolol‐mediated upregulation of Ect2, a guanine nucleotide exchange factor required for cytokinesis (Liu et al., 2019). Consistent in part with the latter paradigm, protein kinase C‐mediated inhibition of cell cycle re‐entry of NNVMs via p38α/β MAPK signaling was associated with the concomitant downregulation of Ect2 mRNA levels (Kebbe et al., 2023). Thus, β‐adrenergic receptor‐mediated attenuation of cell cycle re‐entry of mouse ventricular cardiomyocytes may likewise require p38α/β MAPK signaling to initiate Ect2 mRNA downregulation (Communal et al., 2000; Gao et al., 2010; Sabri et al., 2000). These observations suggest that sympathetic hyperactivation following ischemic damage to the adult mammalian heart may in part limit the appearance of nestin^(+)^‐ventricular cardiomyocytes and concomitantly suppress the cell cycle re‐entry of nestin^(−)^‐ and/or nestin^(+)^‐ventricular cardiomyocytes via p38α/β MAPK signaling (El‐Helou et al., 2008; Engel et al., 2005, 2006; Gan et al., 2020; Hertig et al., 2019; Liu et al., 2019). Thus, the present study tested the hypothesis that sympathetic stimulation of the β_1_/β_2_‐adrenergic receptor and subsequent recruitment of p38α/β MAPK suppresses the cell cycle re‐entry of both subpopulations of neonatal rat ventricular cardiomyocytes.

## 2 METHODS

### 2.1 Ethics approval

The use and care of laboratory rodents was according to the Canadian Council for Animal Care and the protocol (Régénération cardiaque chez les rats néonataux et adultes; # 2021‐2895, 2020‐82‐01) was approved by the Animal Care Committee of the Montreal Heart Institute. The study was performed and reported in accordance with the ARRIVE guidelines.

### 2.2 Isolation of neonatal ventricular cells from 1‐day‐old Sprague–Dawley rats

Neonatal rat ventricular cardiomyocytes were isolated from a litter of 1‐day‐old Sprague–Dawley rat pups (sex undetermined) (Charles River, Canada) as previously described (Hertig et al., 2019; Kebbe et al., 2023). Neonatal rat pups were anesthetized by hypothermia and sacrificed by decapitation. Ventricular cells were plated at a density of 400 cells/mm^2^ in DMEM‐low glucose (Hyclone Laboratories, Logan, UT) supplemented with 7% heat‐inactivated FBS and 1% penicillin–streptomycin for 48 h, subsequently washed, and maintained in DMEM‐low glucose containing insulin (5 μg/mL), transferrin (5 μg/mL), and selenium (5 ng/mL) (insulin/transferrin/selenium; ITS; BD Bioscience, Bedford, MA) for 24 h prior to the experimental protocol. Ventricular cardiomyocytes isolated from 1‐day‐old neonatal rat hearts represent the predominant population (~75%–80%) of ventricular cells characterized by cardiac troponin‐T staining (Bergeron et al., 2021; El‐Helou et al., 2008; Hertig et al., 2018, 2019; Kebbe et al., 2023; Meus et al., 2017). The remaining modest population of ventricular cells were fibroblasts characterized by the absence of cardiac troponin‐T staining and constitutive alpha 2 type 1 collagen staining (Hertig et al., 2023). Moreover, in contrast to neonatal rat ventricular cardiomyocytes, a subpopulation of neonatal rat ventricular fibroblasts expressed the intermediate filament protein nestin (Hertig et al., 2023). Previous work from our lab identified nestin immunoreactivity in a subpopulation of embryonic ventricular fibroblasts, and expression persisted in the neonatal rat heart (Hertig et al., 2023). Summary of the experimental approaches are highlighted in supplemental figures S1 and S2.

NNVMs were treated for a period of 24 h with phorbol 12, 13‐dibutyrate (PDBu 100 nM; CAS#37558–16‐0, Sigma‐Aldrich, St. Louis, Mo), norepinephrine (NE 1 μM; CAS#329‐56‐6, Sigma‐Aldrich), isoproterenol (ISO 1 μM; CAS#51‐30‐9, Sigma‐Aldrich) with or without the p38α/β MAPK inhibitor SB203580 (10 Μm; CAS#152121‐47‐6, LC Laboratories, Woburn, MA) to assess cell cycle re‐entry and mRNA expression. SB203580 was added 10–15 min prior to the addition of the stimulus. Work from our lab reported that 10 mM SB203580 completely suppressed p38α/β MAPK‐mediated phosphorylation of the downstream target heat shock protein 27 (HSP27) and prevented Yap‐1 phosphorylation in NNVMs following PDBu treatment (Hertig et al., 2018; Meus et al., 2017). The nucleotide analog 5‐bromo‐2′‐deoxyuridine (20 ΜM; CAS#59‐14‐3, Sigma‐Aldrich) was added during the 24‐h treatment of NNVMs.

### 2.3 Western blot

Protein lysates were prepared from neonatal rat ventricular cells and subjected to SDS‐electrophoresis, as previously described (El‐Helou et al., 2008; Hertig et al., 2018, 2019; Kebbe et al., 2023; Meus et al., 2017). Antibodies used include rabbit monoclonal anti‐PKC‐α (1:5000, catalogue #59745; Cell Signaling), rabbit monoclonal anti‐PKC‐δ (1:2000, catalogue #2058; Cell Signaling), rabbit polyclonal anti‐PKC‐ε (1:2000, catalogue #2683; Cell Signaling), and a mouse monoclonal anti‐GAPDH (1:10,000, RRID:AB_437392; Ambion, Austin, TX). To assess the temporal pattern of p38α MAPK recruitment in response to NE (1 μM), phosphorylation of the threonine^180^/tyrosine^182^ residues of p38α MAPK (rabbit polyclonal, 1:500; RRID:AB_330713, Cell Signalling Technology) was determined and normalized to total p38 MAPK protein levels (rabbit polyclonal recognizes p38α MAPK, p38β MAPK and p38γ MAPK; 1:500; RRID:AB_2139682, Cell Signalling Technology). Following overnight incubation at 4°C, the appropriate secondary antibody conjugated to horseradish peroxidase (1:20,000, Jackson Immunoresearch, West Grove, PA) was added, and bands were visualized utilizing the ECL detection kit (Perkin Elmer, Waltham, MA). Films were scanned with Image J software® and the target protein signal was depicted as arbitrary light units.

### 2.4 Immunofluorescence and immunohistochemistry

At the end of the experimental protocol, primary passage neonatal rat ventricular cells were fixed with 2% paraformaldehyde and immunofluorescence was performed as previously described (Hertig et al., 2019; Kebbe et al., 2023). Ventricular cells were treated with 2 M HCl for 15 min to denature DNA to detect nuclear 5‐bromo‐2′‐deoxyuridine incorporation prior to immunofluorescence. Primary antibodies employed include mouse monoclonal anti‐nestin (1:400, RRID:AB_305313; Abcam), rabbit polyclonal anti‐cardiac troponin‐T (1:200, catalogue #ab209813; Cell Signaling), and chicken polyclonal anti‐5‐bromo‐2′‐deoxyuridine (1:500, RRID:AB_10562139; Abcam). The nucleus was identified with 4′,6′‐diamidino‐2‐phenylindole (DAPI, Sigma‐Aldrich) staining. Secondary antibodies used were goat anti‐mouse IgG conjugated to Alexa‐555 or Alexa‐647 (1:600; Life Technologies), goat anti‐rabbit IgG conjugated to Alexa‐488 or Alexa‐647 (1:600; Life Technologies), and a goat anti‐chicken IgG conjugated to Alexa‐488 or Alexa‐555 (1:600; Life Technologies). Immunofluorescence was visualized with a 20× 0.8 NA DIC plan apochromat objective mounted on a Zeiss LSM 710 confocal microscope. Each immunofluorescence image represents a Maximum Intensity Projection (512 × 512 pixels) derived from a Z‐stack consisting of 25 slices/slide. The program ZEN 3.0 SR Black Image Browser (Carl Zeiss Canada, York, Canada) was employed to determine the number of cardiac troponin‐T^(+)^‐NNVMs that re‐entered the S‐phase of the cell cycle and expressed de novo nestin, and the data were normalized to a density of 500 NNVMs, as previously described (El‐Helou et al., 2008; Hertig et al., 2018, 2019; Kebbe et al., 2023; Meus et al., 2017). To assess a hypertrophic response, the surface area (μm^2^) of individual mononucleated NNVMs (100–110 NNVMs per treatment per experiment were assessed from *n* = 4 independent neonatal rat litters) regardless of the absence or presence of nestin was determined with the ZEN 3.0 SR Black Image Browser, as previously reported (Hertig et al., 2019; Kebbe et al., 2023). Lastly, the impact of siRNA‐mediated PKC‐α protein downregulation in cell cycle re‐entry of the S‐phase of neonatal rat ventricular fibroblasts was also determined, and the data were normalized to a total of 100 ventricular fibroblasts, as previously described (Hertig et al., 2023).

### 2.5 siRNA

Neonatal rat ventricular cells were transfected with a siRNA directed against PKC‐α according to the instructions detailed in the OriGene siRNA transfection kit (TT320002, OriGene Biotechnology Company, Rockville, MD). NNVMs plated in 7% FBS for a period of 24 h were transfected with three 27‐mer siRNAs directed against rat PKC‐α (~13 nM of each 27‐mer siRNA for a total concentration of ~40 nM; OriGene, catalogue #SR512339) or a scrambled 27‐mer siRNA (~40 nM; catalogue #SR30004, OriGene), and the transfection procedure was permitted to continue for a period of 24 h. NNVMs were subsequently washed and plated in ITS for 24 h. PKC‐α protein levels were determined by Western blot analysis, and the subsequent impact on cell cycle re‐entry and de novo nestin expression was assessed by Immunofluorescence. In the Western blot assay, PKC‐α protein expression was normalized to β‐actin (rabbit monoclonal anti‐β‐actin, 1:500; Cell Signaling, RRID:AB_2223172). Lastly, to assess the hypertrophic response, the surface area of mononucleated untreated (basal) NNVMs, NNVMs treated with PDBu and the scrambled siRNA, and NNVMs treated with PDBu and the siRNA directed against PKC‐α was determined by immunofluorescence (100–125 NNVMs per experiment; *n* = 4 independent neonatal rat litters).

### 2.6 QPCR

Total RNA extraction of neonatal rat ventricular cardiomyocytes was performed with the NucleoSpin RNA Kit (Macherey‐Nagel, Germany), as previously described (Hertig et al., 2018). Reverse Transcriptase was performed with the High‐Capacity cDNA Reverse Transcription Kit (Thermofisher). Rat primers for each gene were obtained from Thermofisher and Taqman (Thermofisher) used to assess mRNA expression. mRNA levels were normalized to GAPDH and HPRT1 mRNAs (the average of GAPDH and HPRT1 mRNA levels is denoted GEOMEAN). The primers used were Nestin, Rn01455599_g1; NPPA (Atrial natriuretic peptide), Rn00664637_g1; CDKN2a, Rn00580664_m1; Runx1, Rn01645281_m1; ECT2, Rn01457089_m1; Bub1, Rn01484954_m1; REG3β, Rn00583920_m1; GAPDH, Rn01775763_g1; and HPRT1, Rn01527840_m1. Mustn1, Rn00710500_g1; ACTA1, Rn01426628_g1; MYL1, Rn01401546_m1; Myh2, Rn01470656_m1; and Gem1, Rn011439614_m1.

### 2.7 Statistics

Data are presented as the mean ± SD, (*n*) represents the number of independent litters of neonatal rats used for each experimental approach. PKC isoform protein downregulation was evaluated by a Students' unpaired *t*‐test, and a significant difference was determined by a value of *p* < 0.05 (Origin 2016; Northhampton, MA). The density of NNVMs and ventricular fibroblasts that re‐entered the cell cycle, the density of nestin^(+)^‐NNVMs, the surface area of NNVMs, and Western blot data of the siRNA experiments were evaluated by a one‐way ANOVA followed by a Fisher LSD post hoc test, and a significant difference was determined by a value of *p* < 0.05 (Origin 2016). The distribution of NNVMs that re‐entered the cell cycle in the absence or presence of nestin in response to PDBu/SB203580 and NE/SB203580 was determined by a Students' paired *t*‐test, and a significant difference was determined by a value of *p* < 0.05 (Origin). Lastly, mRNA expression in NNVMs was evaluated by a one‐way ANOVA followed by a Fisher LSD post hoc test, and a significant difference was determined by a value of *p* < 0.05 (Origin 2016).

## 3 RESULTS

### 3.1 Cardiac hypertrophy and cycle re‐entry of 1‐day‐old NNVMs treated with norepinephrine and phorbol 12,13‐dibutyrate for 24 h in the absence or presence of SB203580

**FIGURE 1:**
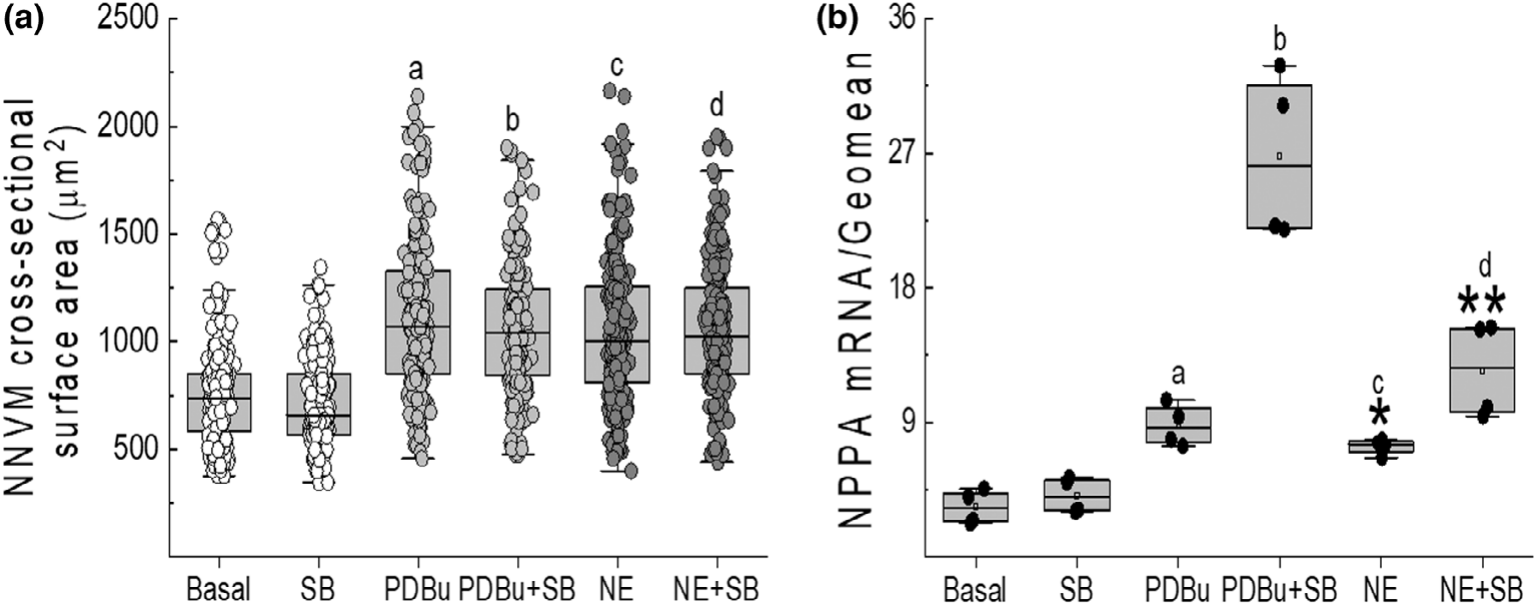
Hypertrophic response of 1‐day‐old neonatal rat ventricular cardiomyocytes (NNVMs). (a) The surface area of individual mononucleated NNVMs (*n* = 4 independent neonatal rat litters) following a 24h treatment with NE (1 μM) or PDBu (100 nM) was significantly increased compared to untreated NNVMs and SB203580 (SB, 10 μM) treatment was without effect. Data were analyzed by a one‐way ANOVA followed by a Fisher LSD post hoc test and statistical difference was observed between PDBu versus Basal (A; *p* = 0.001), PDBu+SB versus SB (B; *p* = 0.001), NE versus basal (C; *p* = 0.001), and NE + SB versus SB (D; *p* = 0.001). (b) In parallel, norepinephrine (NE, 1 μM) and PDBu (100 nM) treatment of NNVMs (*n* = 4 independent neonatal rat litters) for 24 h significantly increased NPPA (atrial natriuretic peptide) mRNA levels and expression was potentiated following SB203580 treatment. mRNA data was normalized to GAPDH and HPRT‐1 mRNA levels (referred to as GEOMEAN). Data were analyzed by a one‐way ANOVA followed by a Fisher LSD post hoc test and statistical difference was observed between PDBu versus basal (A; *p* = 0.024), PDBu+SB versus PDBu (B; *p* = 2e‐6), NE versus basal (C; *p* = 0.008), and NE + SB versus NE (D; *p* = 0.002).

**FIGURE 2:**
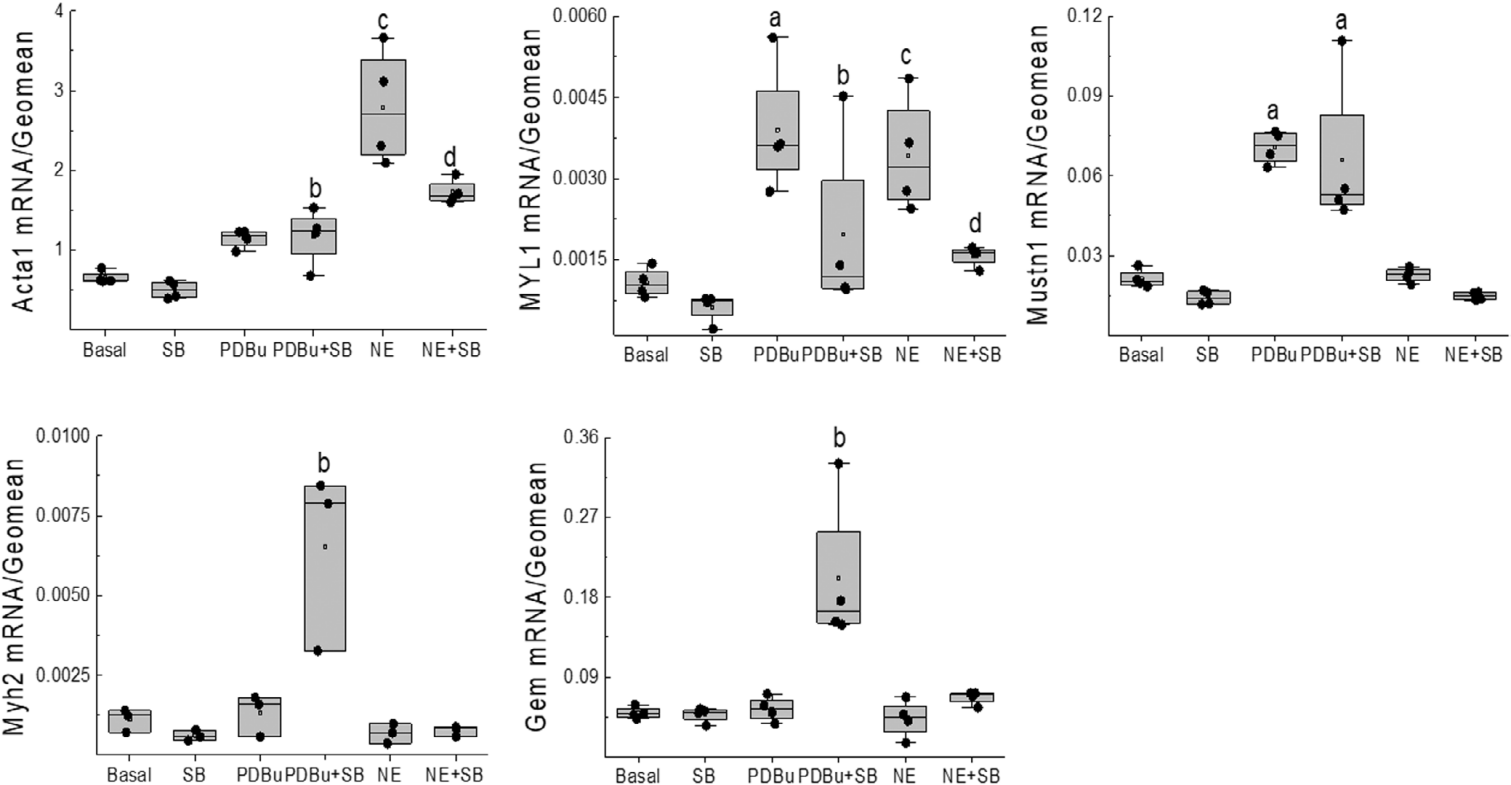
The transcript profile of skeletal muscle genes. The 24h treatment of NNVMs (*n* = 4 independent neonatal rat litters) with PDBu (100 nM) significantly increased skeletal myosin light chain 1 (MYL1) (a; *p* = 7e‐4 vs. basal) and musculoskeletal embryonic nuclear protein 1 (Mustn1) mRNA levels (a; *p* = 3e‐5 vs. basal) whereas the mRNA expression of skeletal α‐Actin (ACTA1) (*p* = 0.06), skeletal muscle myosin heavy chain 2 (Myh2) (*p* = 0.84), and skeletal muscle‐specific GTP binding protein (Gem1) (*p* = 0.87) remained unchanged as compared to untreated (basal) NNVMs. PDBu/SB203580 (SB, 10 μM) treatment significantly inhibited the mRNA upregulation of MYL1 (b; *p* = 0.012 vs. PDBu) whereas Mustn1 mRNA levels remained unchanged as compared to PDBu (*p* = 0.60 vs. PDBu) but was significantly increased versus untreated or SB203580‐treated NNVMs (a; *p* = 1e‐5 vs. basal/SB‐treated). Moreover, PDBu/SB203580 treatment of NNVMs significantly increased ACTA1 (b; *p* = 0.013 vs. SB‐treated), Myh2 (b; *p* = 6e‐5 vs. SB‐treated), and Gem1 mRNA levels (b; *p* = 1e‐5 vs. SB‐treated) as compared to SB203580‐treated NNVMs. Norepinephrine (NE, 1 μM) stimulation of NNVMs for 24h significantly increased ACTA1 (c; *p* = 7e‐8 vs. basal) and MYL1 (c; *p* = 0.003 vs. basal) mRNA levels whereas Myh2 (*p* = 0.66), Mustn1 (*p* = 0.88), and Gem1 (*p* = 0.80) mRNA expression was unchanged as compared to untreated (basal) NNVMs. SB203580 treatment of NE‐stimulated NNVMs significantly inhibited the mRNA upregulation of ACTA1 (d; *p* = 3e‐4 vs. NE) and MYL1 (d; *p* = 0.015 vs. NE). mRNA data was normalized to GAPDH and HPRT‐1 mRNA levels (referred to as GEOMEAN) and data were analyzed by a one‐way ANOVA followed by a Fisher LSD post hoc test.

NE (1 μM) treatment for 24 h significantly increased the surface area of a subpopulation of cardiac troponin‐T^(+)^‐NNVMs, translating to a significant hypertrophic response accompanied by a robust increase in NPPA mRNA levels as compared to untreated NNVMs (Figure 1a,b). NE‐induced NPPA mRNA expression was associated with the concomitant upregulation of hypertrophic‐associated skeletal muscle genes, as sympathetic stimulation of NNVMs increased the mRNA levels of skeletal α‐actin (ACTA1) and skeletal myosin light chain 1 (MYL1) (Paradis et al., 1996). By contrast, NE stimulation of NNVMs did not alter the mRNA levels of musculoskeletal embryonic nuclear protein 1 (Mustn1), skeletal muscle myosin heavy chain 2 mRNA (Myh2), or skeletal muscle‐specific GTP binding protein (Gem1) (Figure 2).

**FIGURE 3:**
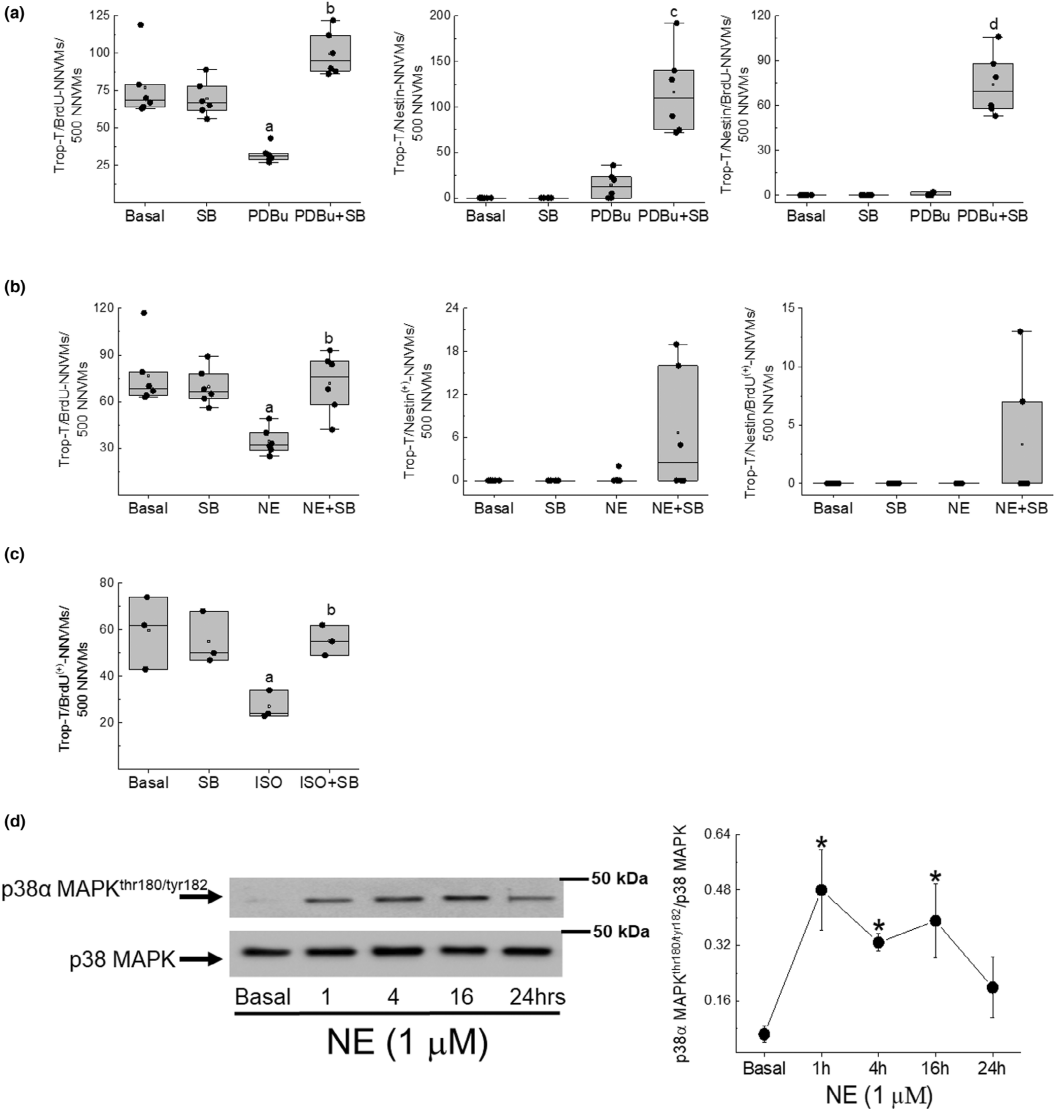
Quantitative assessment of cell cycle re‐entry of 1‐day‐old neonatal rat ventricular cardiomyocytes (NNVMs) and recruitment of p38α/β MAPK signaling in response to sympathetic stimulation. (a) Nuclear incorporation of the nucleotide analog 5‐bromo‐2′‐deoxyuridine (BrdU; *n* = 8 independent neonatal rat litters) was similar between untreated (basal) and SB203580‐treated (SB, 10 μM) NNVMs. PDBu (100 nM) treatment NNVMs for 24 h significantly reduced 5‐bromo‐2′‐deoxyuridine (A; *p* = 5e‐4 vs. basal). In the presence of SB203580, PDBu‐mediated inhibition of 5‐bromo‐2′‐deoxyuridine incorporation was significantly reversed (B; *p* = 9e‐4 vs. PDBu). PDBu/SB203580 treatment led to the de novo nestin expression (C; *p* = 9e‐4 vs. PDBu) and increased the density that incorporated BrdU (D; *p* = 4e‐9 vs. PDBu). (b) Norepinephrine (NE, 1 μM) treatment NNVMs for 24 h significantly reduced the nuclear incorporation of 5‐bromo‐2′‐deoxyuridine (A; *p* = 2e‐4 vs. basal). In the presence of SB203580, NE‐mediated inhibition of 5‐bromo‐2′‐deoxyuridine incorporation was significantly reversed (B; *p* = 5e‐4 vs. PDBu). By contrast, in the presence of NE/SB203580, the cell cycle re‐entry of the predominant subpopulation of NNVMs was not associated with de novo nestin expression. Data were analyzed by a one‐way ANOVA followed by a Fisher LSD post hoc test. (c) The treatment of NNVMs (*n* = 3 independent neonatal rat litters) with isoproterenol (ISO, 1 μM) for 24 h significantly reduced nuclear incorporation of 5‐bromo‐2′‐deoxyuridine (A; *p* = 0.001 vs. basal) and SB203580 (SB; 10 μM) treatment reversed the inhibitory effect on cell cycle re‐entry (B; *p* = 0.01 vs. basal) in the absence of de novo nestin expression. (d) The treatment of NNVMs (*n* = 3 independent neonatal rat litters) with norepinephrine (NE, 1 μM) led to a rapid significant increase in the phosphorylation of threonine^180^/tyrosine^182^ residues of p38α MAPK at 1 h (*p = 0.004 vs. basal), 4 h (*p = 0.042 vs. basal) and persisted for at least 16 h (*p = 0.017 vs. basal). p38α MAPK phosphorylation was normalized to the total p38 MAPK protein expression. Data were analyzed by a one‐way ANOVA followed by a Fisher LSD post hoc test.

**FIGURE 4:**
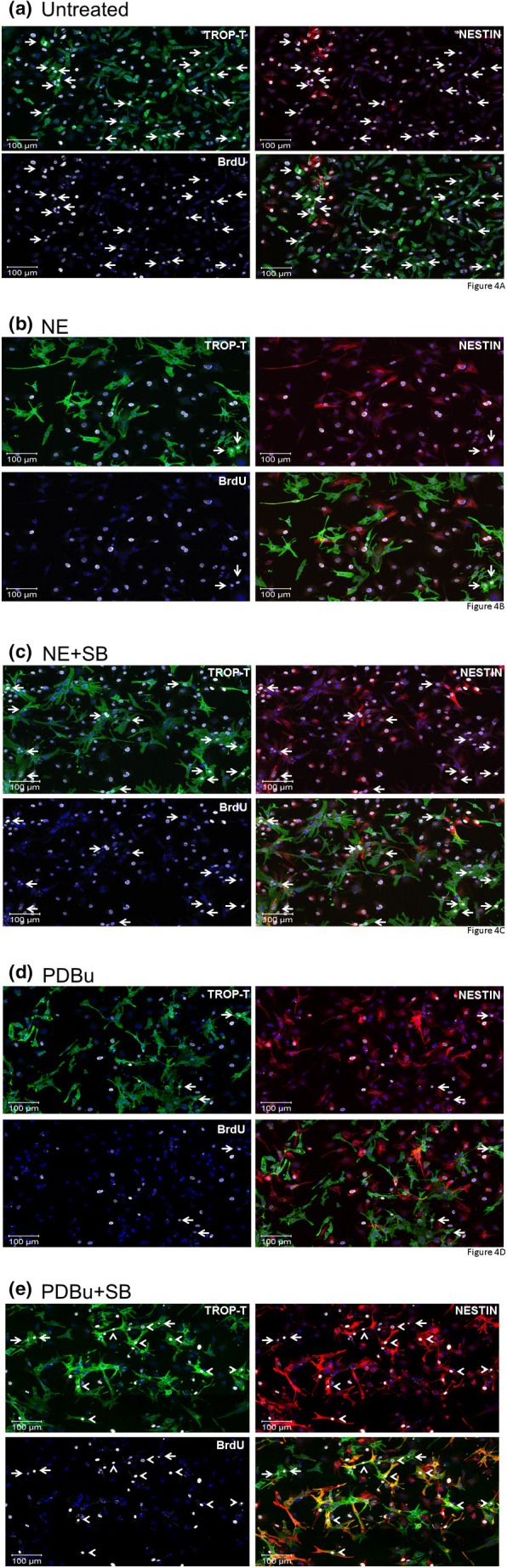
Qualitative assessment of cell cycle re‐entry of 1‐day‐old neonatal rat ventricular cardiomyocytes (a) In untreated NNVMs, 5‐bromo‐2′‐deoxyuridine incorporation (gray fluorescence) was identified in cardiac troponin‐T^(+)^‐NNVMs (TROP‐T, green fluorescence; indicated by arrow). Nestin (red fluorescence) staining was absent in cardiac troponin‐T^(+)^‐NNVMs whereas immunoreactivity was detected in ventricular fibroblasts lacking expression of the cardiomyocyte marker. (b) In norepinephrine (NE, 1 μM)‐treated NNVMs, the density of cardiac troponin‐T^(+)^‐NNVMs (green fluorescence; indicated by arrow) that incorporated 5‐bromo‐2′‐deoxyuridine (gray fluorescence) was significantly lower versus untreated NNVMs. (c) In NE/SB203580‐treated NNVMs, the density of cardiac troponin‐T^(+)^‐NNVMs (green fluorescence; indicated by arrow) that incorporated 5‐bromo‐2′‐deoxyuridine (gray fluorescence) was significantly increased versus NE‐treated NNVMs. Nestin (red fluorescence) immunoreactivity was absent in NE‐treated NNVMs but staining was detected in ventricular fibroblasts. (d) In PDBu (100 nM)‐treated NNVMs, the density of cardiac troponin‐T^(+)^‐NNVMs (green fluorescence; indicated by arrow) that incorporated 5‐bromo‐2′‐deoxyuridine (gray fluorescence) was reduced versus untreated NNVMs. Nestin (red fluorescence) immunoreactivity was predominantly detected in ventricular fibroblasts. (e) In PDBu/SB203580‐treated NNVMs, the density of cardiac troponin‐T^(+)^‐NNVMs (green fluorescence) that incorporated 5‐bromo‐2′‐deoxyuridine (gray fluorescence) was significantly increased and detected in two distinct subpopulations distinguished by the absence (indicated by arrow) or presence of de novo nestin expression (indicated by arrowhead). The nucleus was identified with 4′,6′‐diamidino‐2‐phenylindole (DAPI) staining (blue fluorescence).

In untreated NNVMs, nuclear incorporation of 5‐bromo‐2′‐deoxyuridine was evident in the subpopulation that lacked de novo nestin expression and occurred in the absence of p38α/β MAPK inhibition (Figures 3a and 4a). NE‐stimulated hypertrophy coincided with a significant reduction in the density of a subpopulation of NNVMs that incorporated 5‐bromo‐2′‐deoxyuridine, highlighting the concomitant suppression of cell cycle re‐entry (Figures 3a and 4b). The latter response occurred at least in part via the β_1_/β_2_‐adrenergic receptor as the nonselective β‐adrenergic agonist isoproterenol (ISO, 1 μM) significantly reduced the density of NNVMs that incorporated 5‐bromo‐2′‐deoxyuridine as compared to untreated NNVMs (Figure 3c).

**FIGURE 5:**
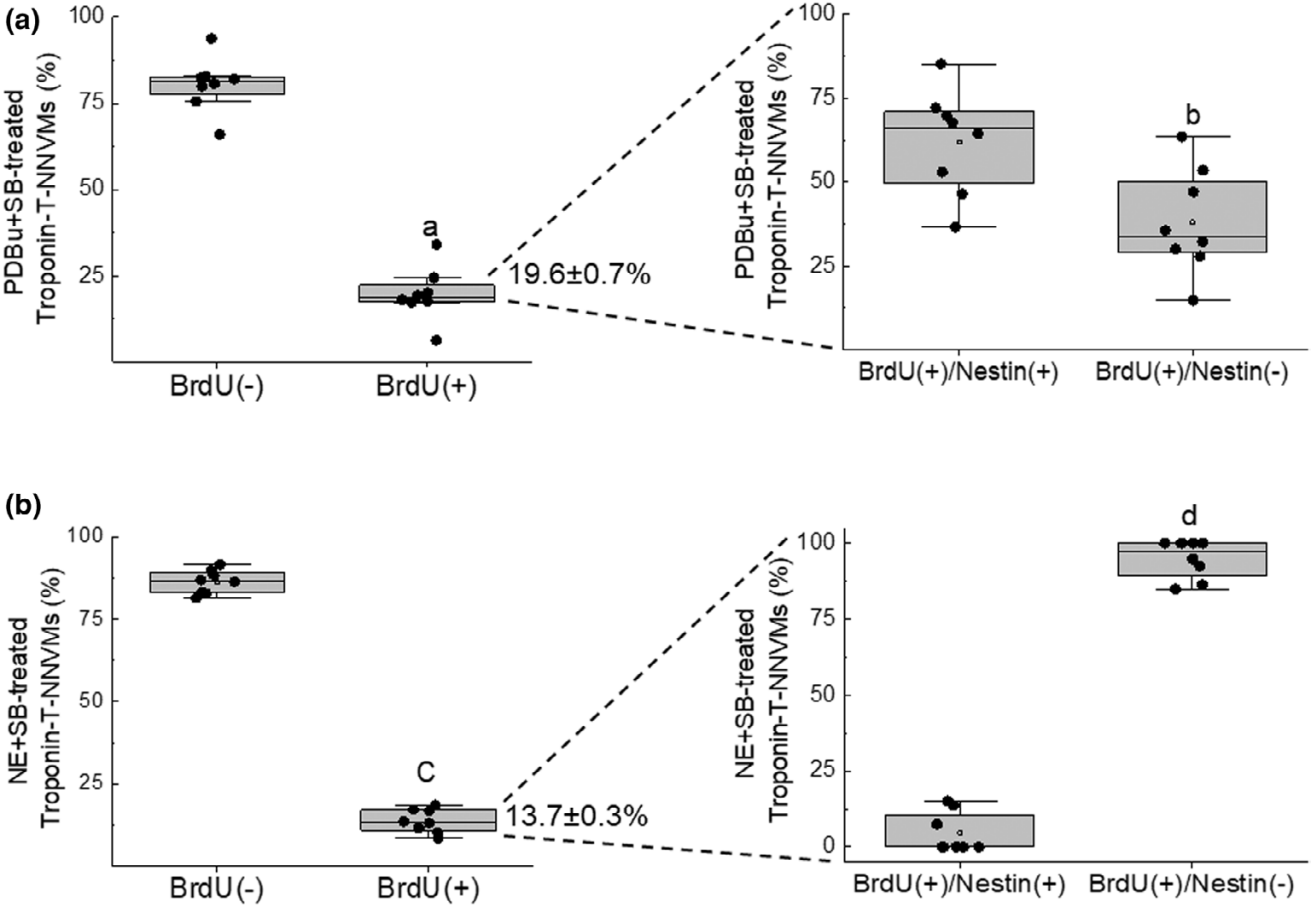
Distribution of 1‐day‐old neonatal rat ventricular cardiomyocytes (NNVMs) that re‐entered the cell cycle. (a) A modest population (~20%) of NNVMs re‐entered the S‐phase of the cell cycle following a 24h treatment with PDBu (100 nM) and SB203580 (SB, 10 μM) (A; *p* = 1e‐10 vs. NNVMs that did not incorporate BrdU). Within the population of NNVMs that re‐entered the S‐phase, the predominant subpopulation was distinguished by de novo nestin expression (B; *p* = 0.0088 vs. nestin^(−)^‐NNVMs) whereas the minor subpopulation lacked expression of the intermediate filament protein. (b) Norepinephrine (NE, 1 μM) and SB203580 (SB, 10 μM) treatment of NNVMs for 24 h induced 5‐bromo‐2′‐deoxyuridine incorporation in a modest subpopulation (~14%) (C; *p* = 7e‐16 vs. NNVMs that did not incorporate BrdU). Within the population that re‐entered the cell cycle, the predominant subpopulation lacked de novo nestin expression (D; *p* = 1e‐13 vs. nestin^(+)^ NNVMs). Data were analyzed by a Students paired *t*‐test.

The stimulation of NNVMs (*n* = 3 independent neonatal rat litters) with NE (1 μM) led to the rapid phosphorylation of the threonine^180^ and tyrosine^182^ residues of p38α MAPK, and phosphorylation remained significantly elevated 16 h after initial treatment (Figure 3d). SB203580 (10 μM) treatment failed to attenuate the NE‐mediated increase in the surface area of NNVMs and significantly potentiated NPPA mRNA levels, as compared to NE treatment alone (Figure 1a,b). NE‐mediated upregulation of ACTA1 and MYL1 mRNAs was significantly reduced following SB203580 treatment (Figure 2). By contrast, SB203580 treatment reversed NE‐mediated suppression of cell cycle re‐entry, as the density of NNVMs that incorporated 5‐bromo‐2′‐deoxyuridine was significantly increased as compared to NE treatment alone (Figures 3a and 4c). SB203580 (10 μM) administration to ISO‐stimulated NNVMs likewise promoted re‐entry into the cell cycle, as the density that incorporated 5‐bromo‐2′‐deoxyuridine was significantly higher versus ISO treatment alone (Figure 3c). A salient feature of these data is that only ~13% of the total density of NNVMs were associated with 5‐bromo‐2′‐deoxyuridine incorporation following NE/SB203580 or ISO/SB203580 treatment (Figure 5a). Within the density of NNVMs that incorporated 5‐bromo‐2′‐deoxyuridine following NE/SB203580 treatment, a paucity was associated with de novo nestin expression, whereas staining of the intermediate filament protein was detected in ventricular fibroblasts (Figures 2a and 3c). A detailed analysis of the total density of NNVMs that incorporated 5‐bromo‐2′‐deoxyuridine after NE/SB203580 treatment (13.7 ± 0.3%) revealed that the majority that re‐entered the cell cycle (~97%) were not associated with de novo nestin expression (Figures 3c, 4c, and 5a). Consistent with the latter data, ISO/SB203580‐mediated re‐entry into the cell cycle of NNVMs occurred in the absence of de novo nestin expression (*data not shown*).

The treatment of NNVMs for 24 h with the protein kinase C activator phorbol 12,13‐dibuytrate (PDBu; 100 nM) induced a hypertrophic response concomitant with a significant upregulation of NPPA mRNA levels (Figure 1a,b). Furthermore, PDBu‐induced NNVM hypertrophy concomitantly upregulated the mRNA levels of MYL1 and Mustn1 whereas Myh2, Gem1, and ACTA1 mRNA expression was not significantly changed (Figure 2). SB203580 treatment of PDBu‐stimulated NNVMs did not reverse the hypertrophic response, and NPPA mRNA levels were further potentiated compared to PDBu treatment alone (Figure 2). In parallel, SB203580 treatment of PDBu‐stimulated NNVMs significantly attenuated the mRNA upregulation of MYL1 whereas SB20350 failed to inhibit PDBu‐mediated increase of Mustn1 mRNA levels (Figure 2). However, SB203580 treatment of PDBu‐treated NNVMs significantly increased the mRNA expression of ACTA1, MYH2, and Gem1 as compared to PDBu‐treated NNVMs (Figure 2).

Previous studies have reported that a 3‐day treatment of NNVMs with PDBu alone had no effect on cell cycle re‐entry (El‐Helou et al., 2008; Hertig et al., 2019; Kebbe et al., 2023). However, in the present study, PDBu treatment for 24 h significantly reduced 5‐bromo‐2′‐deoxyuridine incorporation in a subpopulation of NNVMs distinguished by the absence of de novo nestin expression (Figures 3b and 4d). Moreover, PDBu treatment alone modestly increased the density of NNVMs associated with de novo nestin expression but failed to induce 5‐bromo‐2′‐deoxyuridine incorporation (Figures 3b and 4d). PDBu‐mediated inhibition of cell cycle re‐entry of a subpopulation of NNVMs was completely reversed with SB203580 treatment as the density that incorporated 5‐bromo‐2′‐deoxyuridine was increased and significantly greater than PDBu treatment alone (Figures 3b and 4e). Akin to that observed with NE/SB203580 treatment, only a modest subpopulation of NNVMs re‐entered the cell cycle (~20%) following PDBu/SB203580 treatment (Figure 5b). The predominant subpopulation of NNVMs that incorporated 5‐bromo‐2′‐deoxyuridine (~65%) after PDBu/SB203580 treatment was associated with de novo nestin expression (Figures 3b, 4e, and 5b). In addition, and analogous to that observed following NE/SB203580 administration, a minor subpopulation of NNVMs (~35%) that re‐entered the cell cycle after PDBu/SB203580 treatment was characterized by the absence of de novo nestin expression (Figures 3b, 4e, and 5b).

### 3.2 mRNA profile of genes implicated in cell cycle re‐entry

**FIGURE 6:**
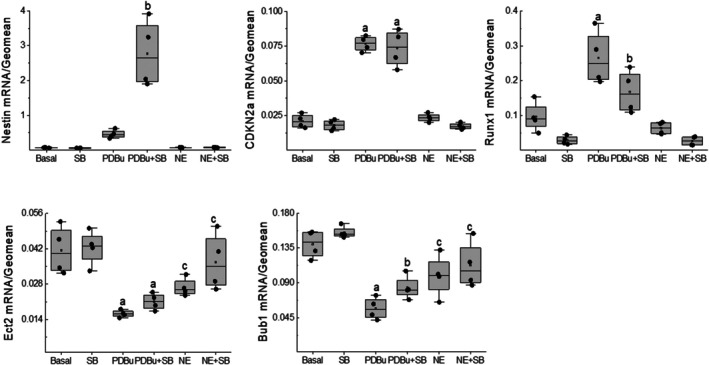
The transcript profile of cell cycle genes. The 24h treatment of neonatal ventricular cells (*n* = 4 independent neonatal rat litters) with PDBu (100 nM) modestly but nonsignificantly increased nestin mRNA levels (*p* = 0.28) whereas Runx1 (a; *p* = 2e‐06 vs. basal) and CDKN2a (a; *p* = 3e‐07 vs. basal) mRNA levels were upregulated. By contrast, PDBu stimulation significantly reduced Ect2 (a; *p* = 2e‐05 vs. basal) and Bub1 (a; *p* = 3e‐06 vs. basal) mRNA expression as compared to untreated (basal) ventricular cells. SB203580 (SB, 10 μM) administration to PDBu‐treated ventricular cells potentiated nestin mRNA expression (b; *p* = 2e‐05 vs. PDBu), partially and significantly reduced elevated Runx1 (b; *p* = 0.0046 vs. PDBu) mRNA levels whereas CDKN2a mRNA expression remained increased and significantly greater (a; *p* = 3e‐07 vs. basal/SB203580) compared to untreated and SB203580‐treated ventricular cells. SB203580 administration to PDBu‐treated ventricular cells partially and significantly reversed the mRNA downregulation of Bub1 (b; *p* = 0.02 vs. PDBu) whereas Ect2 mRNA levels remained significantly reduced versus untreated and SB203580‐treated ventricular cells (a; *p* = 2e‐05 vs. basal/SB). The 24h treatment of ventricular cells with norepinephrine (NE, 1 μM) selectively downregulated Ect2 (c; *p* = 1.5e‐04 vs. basal) and Bub1 (c; *p* = 4e‐05 vs. basal) mRNA levels whereas nestin (*p* = 0.82), Runx1 (*p* = 0.76), and CDKN2a (*p* = 0.74) mRNA expression remained unchanged as compared to untreated (basal) ventricular cells. SB203580 administration to NE‐treated ventricular cells failed to reverse ECT2 (c; *p* = 2.5e‐04 vs. basal/SB203580) and Bub1 (c; *p* = 0.001 vs. basal/SB203580) mRNA downregulation. Data were analyzed by a one‐way ANOVA followed by a Fisher LSD post hoc test.

**FIGURE 7:**
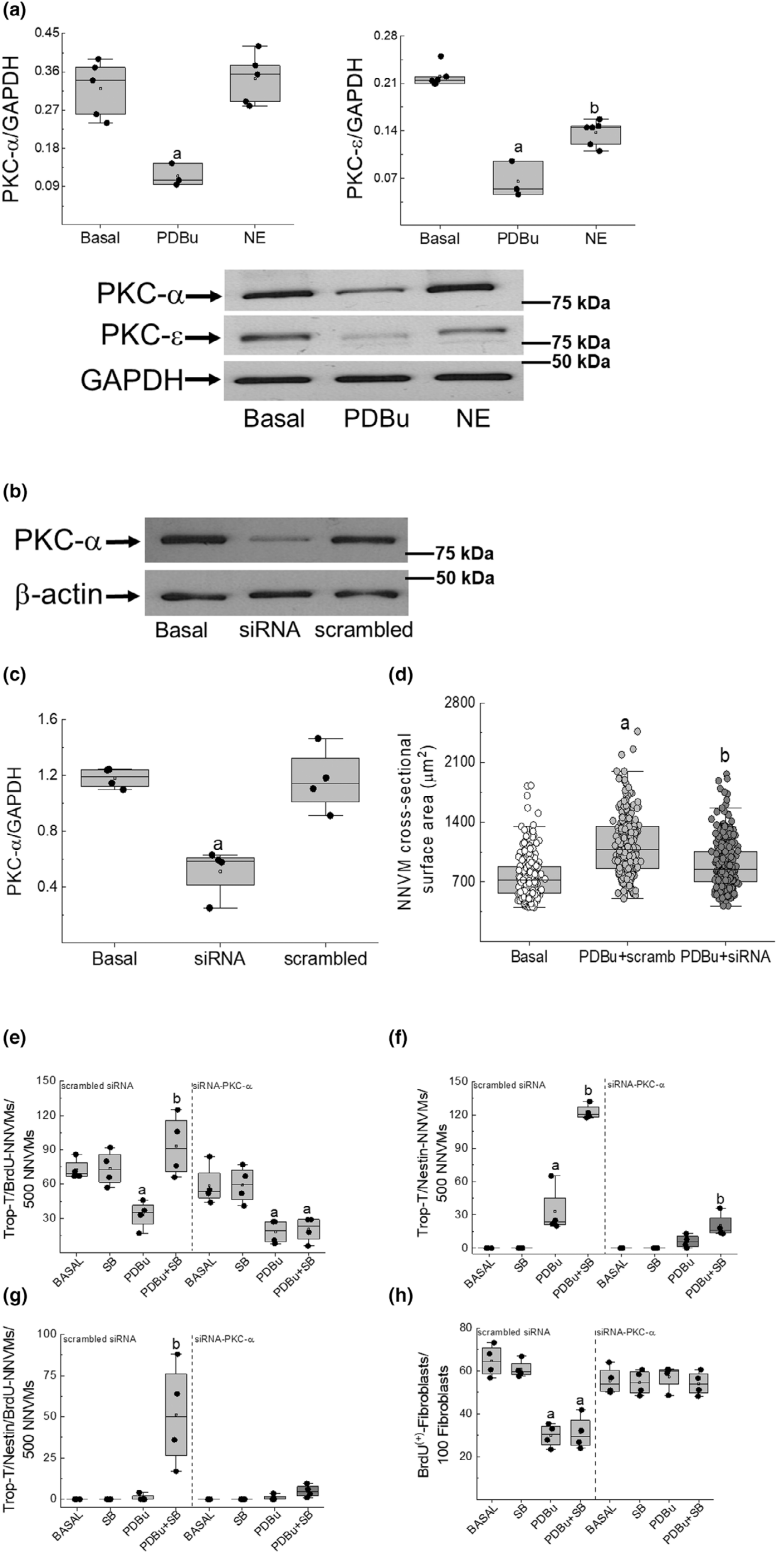
PKC isoform regulation in neonatal rat ventricular in response to NE and PDBu. (a) PDBu (100 nM) stimulation of ventricular cells (*n* = 3 independent neonatal rat litters) for 24 h robustly downregulated PKC‐α (a; *p* = 6e‐4 vs. basal), PKC‐ε (a; *p* = 6e‐8 vs. basal), and PKC‐δ (data not shown) protein levels versus untreated (basal) ventricular cells. By contrast, norepinephrine (NE, 1 μM) stimulation (*n* = 5–6 independent neonatal rat litters) for 24 h downregulated PKC‐ε (b; *p* = 6e‐6 vs. basal) protein levels whereas PKC‐α (*p* = 0.52) and PKC‐δ (data not shown) expression remained unchanged versus untreated (basal) ventricular cells. Data was analyzed by a Students' unpaired *t*‐test. (b, c) A 24h treatment of ventricular cells with a siRNA (*n* = 4 independent neonatal rat litters) directed against PKC‐α significantly reduced protein levels (a; *p* = 3.8e‐4 vs. basal) as compared to untreated (basal) ventricular cells. By contrast, ventricular cells treated with a scrambled siRNA had no effect on PKC‐α protein expression (*p* = 0.91). Data were analyzed by a one‐way ANOVA followed by a Fisher LSD post hoc test and PKC‐α protein levels were normalized to β‐Actin. (d) As indicated in Figure 1a, PDBu (100 nM) treatment of NNVMs for a period of 24 h transfected with the scrambled siRNA (scramb) increased the surface area of a subpopulation of NNVMs (a; *p* = 5e‐7 versus basal) as compared to untreated (basal) NNVMs. siRNA‐mediated downregulation of PKC‐α protein levels of significantly attenuated the surface area (b; *p* = 1e‐7 vs. PDBu+scrambled siRNA) of a subpopulation of transfected NNVMs as compared to PDBu‐treated NNVMs transfected with the scrambled siRNA. Data were analyzed by a one‐way ANOVA followed by a Fisher LSD post hoc test. (e) Following the transfection of NNVMs with the scrambled siRNA (*n* = 4 independent neonatal rat litters), PDBu (100 nM) stimulation (a; *p* = 0.026 vs. basal) significantly reduced 5‐bromo‐2′‐deoxyuridine (BrdU) incorporation in the subpopulation of nestin^(−)^‐NNVMs and SB203580 (SB; 10 μM) treatment reversed the response (b; *p* = 0.007 versus PDBu). Despite siRNA‐mediated downregulation of PKC‐α protein levels, PDBu‐mediated inhibition of 5‐bromo‐2′‐deoxyuridine incorporation (a; *p* = 0.003 vs. basal) in NNVMs persisted whereas re‐entry into the cell cycle following SB203580 treatment was prevented and remained significantly lower versus basal (a; *p* = 0.006 versus basal). (f, g) PDBu (100 nM) treatment of NNVMs transfected with the scrambled siRNA (*n* = 4 independent neonatal rat litters) was associated with the modest appearance of a subpopulation of NNVMs characterized by de novo nestin expression (a; *p* = 0.001 vs. basal) but failed to re‐enter the cell cycle. Following SB203580 treatment, PDBu stimulation of NNVMs transfected with the scrambled siRNA significantly increased the density associated with de novo nestin expression (b; *p* = 9e‐7 vs. PDBu) and the density of nestin^(+)^‐NNVMs that incorporated BrdU (b; *p* = 6e‐4 vs. SB). The transfection of NNVMs with an siRNA directed against PKC‐α (*n* = 4 independent neonatal rat litters) led to the modest appearance of a subpopulation of nestin^(+)^‐NNVMs in response to PDBu that was not significantly different (*p* = 0.19) from untreated NNVMs. In PKC‐α depleted NNVMs, PDBu/SB203580 mediated de novo nestin expression in a subpopulation of NNVMs was markedly attenuated but was modestly and significantly greater that PDBu‐treated NNVMs (b; *p* = 0.017 vs. PDBu‐treated NNVMs). Moreover, re‐entry into the S‐phase of the cell cycle was suppressed in the modest subpopulation of nestin^(+)^‐NNVMs following PDBu/SB203580 treatment of PKC‐α depleted NNVMs. Data were analyzed by a one‐way ANOVA followed by a Fisher LSD post hoc test. (h) PDBu (100 nM) treatment of scrambled‐siRNA transfected ventricular fibroblasts (*n* = 4 independent neonatal rat litters) significantly attenuated the nuclear incorporation of 5‐bromo‐2′‐deoxyuridine (BrdU) (a; *p* = 2e‐5 vs. basal) as compared to untreated (basal) ventricular fibroblasts. SB203580 treatment of ventricular fibroblasts transfected with the scrambled‐siRNA in the presence of PDBu failed to reverse the inhibition of cell cycle re‐entry (*p* = 0.78) and remained significantly lower versus untreated ventricular fibroblasts (a; *p* = 7e‐7 vs. basal). siRNA‐mediated PKC‐α protein downregulation in ventricular fibroblasts reversed PDBu‐ (*p* = 0.67 vs. basal) and PDBu/SB203580‐mediated inhibition (*p* = 0.76 vs. basal) of 5‐bromo‐2′‐deoxyuridine incorporation as compared to untreated ventricular fibroblasts. Data were analyzed by a one‐way ANOVA followed by a Fisher LSD post hoc test.

Following a 24h treatment of neonatal rat ventricular cells, PDBu‐mediated inhibition of cell cycle re‐entry of NNVMs was associated with the upregulation of Runx1 and CDKN2a transcripts, whereas Ect2 mRNA levels were markedly reduced as compared to untreated ventricular cells (Figure 7). Furthermore, mRNA levels of the mitotic checkpoint serine/threonine kinase Bub1 were significantly decreased following a 24h treatment of neonatal rat ventricular cells with PDBu as compared to untreated ventricular cells (Figure 6). The appearance and subsequent re‐entry into the S‐phase of the cell cycle of nestin^(+)^‐NNVMs following the PDBu/SB203580 treatment (Figure 4b) were reaffirmed via nestin mRNA upregulation (Figure 6). PDBu/SB203580 treatment of neonatal rat ventricular cells for 24 h failed to inhibit CDKN2a mRNA upregulation but partially and significantly attenuated Runx1 transcript upregulation as compared to PDBu (Figure 6). Furthermore, PDBu/SB203580 treatment of neonatal rat ventricular cells for 24 h significantly attenuated Bub1 mRNA downregulation, whereas a modest reversal of Ect2 mRNA downregulation was also observed but did not reach statistical significance as compared to PDBu (Figure 6).

Following a 24h treatment of neonatal rat ventricular cells with NE, the significant reduction of 5‐bromo‐2′‐deoxyuridine incorporation in NNVMs was not associated with Runx1 or CDKN2a mRNA upregulation (Figure 6). By contrast, NE treatment significantly reduced ECT2 and Bub1mRNA levels as compared to untreated ventricular cells (Figure 6). The observed cell cycle re‐entry of NNVMs following a 24h treatment with NE/SB203580 failed to reverse ECT2 and Bub1 mRNA downregulation or alter the transcript expression of nestin, Runx1, or CDKN2a in ventricular cells (Figure 6).

### 3.3 The role of distinct PKC isoforms in the cell cycle re‐entry of 1‐day‐old NNVMs

As previously reported, chronic phorbol ester stimulation of neonatal rat ventricular cells recruited both conventional and novel PKC isoforms, as reflected by subsequent protein downregulation (Kebbe et al., 2023). Consistent with the latter premise, the 24h treatment of neonatal rat ventricular cells with PDBu recruited conventional and novel PKC isoforms, as depicted by robust downregulation of PKC‐α, PKC‐ε, and PKC‐δ (data not shown) protein levels (Figure 7a) (Kebbe et al., 2023). By contrast, NE (1 μM) treatment of neonatal rat ventricular cells for 24 h preferentially recruited the PKC‐ε isoform, depicted by significant protein downregulation, whereas PKC‐α (Figure 7a) and PKC‐δ protein expression (untreated, 0.36 ± 0.065 vs. NE, 0.38 ± 0.098; *n* = 4 independent litters; normalized to GAPDH) was not significantly changed (Figure 7a). siRNA targeting of PKC‐α markedly reduced protein levels of the serine/threonine kinase compared to neonatal rat ventricular cells treated with scrambled‐siRNA (Figure 7b,c). In parallel, siRNA‐mediated reduction of PKC‐α protein levels significantly reduced PDBu‐mediated hypertrophic response of NNVMs (Figure 7d) as compared to NNVMs treated with scrambled‐siRNA (Figure 7d). With regard to cell cycle re‐entry, PDBu treatment of NNVMs for 24 h significantly reduced 5‐bromo‐2′‐deoxyuridine incorporation, despite siRNA‐mediated downregulation of PKC‐α protein content as compared to NNVMs treated with scrambled siRNA (Figure 7e). In the presence of SB203580, PDBu stimulation of NNVMs was unable to restore cell cycle re‐entry into the S‐phase following siRNA‐mediated PKC‐α protein downregulation (Figure 7e). The latter response was directly attributed to the significant attenuation of de novo nestin expression, thereby abolishing re‐entry into the S‐phase of the cell cycle of this particular subpopulation of NNVMs (Figure 7f,g). As indicated in the Methods section, the isolation/culturing of neonatal ventricular cardiomyocytes was associated with the concomitant appearance of a modest population of ventricular fibroblasts. Work from our lab reported that PDBu treatment of neonatal rat ventricular fibroblasts attenuated cell cycle re‐entry (Hertig et al., 2023). Thus, the potential suppressive role of PKC‐α in the cell cycle re‐entry of ventricular fibroblasts was likewise examined following siRNA‐mediated downregulation of the serine/threonine kinase. In scrambled‐siRNA treated neonatal rat ventricular cells, PDBu stimulation for 24 h significantly reduced 5‐bromo‐2′‐deoxyuridine as compared to untreated fibroblasts, and the response persisted in the presence of SB203580 (Figure 7h). By contrast, siRNA‐mediated PKC‐α protein downregulation abolished PDBu and PDBu/SB203580‐mediated inhibition of 5‐bromo‐2′‐deoxyuridine incorporation in ventricular fibroblasts (Figure 7h).

## 4 DISCUSSION

Previous studies have reported that sympathetic stimulation of the α_1_‐adrenergic receptor selectively increased the cell size of neonatal and adult rodent ventricular cardiomyocytes and induced a pattern of gene expression associated with a hypertrophic response (Al Katat et al., 2022; Cotecchia et al., 2015; Long et al., 1989; Paradis et al., 1996). In the present study, a subpopulation of NNVMs treated with norepinephrine translated to a hypertrophic response and concomitantly induced NPPA, ACTA1, and MYL1 mRNA expression. By contrast, sympathetic stimuli acting via the β_1_/_2_‐adrenergic receptor attenuated the proliferative response of embryonic and juvenile mouse ventricular cardiomyocytes (Feridooni et al., 2017; Liu et al., 2019; Sakabe et al., 2022). Work from our lab and others revealed that p38α/β MAPK signaling in rodent and zebrafish ventricular cardiomyocytes suppressed cell cycle re‐entry, and sympathetic stimulation of β_1_/_2_‐adrenergic receptors recruited the serine/threonine kinase (Bergeron et al., 2021; Communal et al., 2000; Engel et al., 2005, 2006; Gao et al., 2010; Hertig et al., 2018, 2019; Jopling et al., 2012; Kebbe et al., 2023; Meus et al., 2017; Sabri et al., 2000). In the present study, norepinephrine treatment of NNVMs increased the phosphorylation state of p38α MAPK and concomitantly attenuated nuclear incorporation of 5‐bromo‐2′‐deoxyuridine. The latter response was mimicked by the nonselective agonist isoproterenol, supporting the preferential inhibitory role of β_1_/_2_‐adrenergic receptors in the cell cycle re‐entry of NNVMs. Furthermore, the treatment of NNVMs with the p38α/β MAPK inhibitor SB203580 completely reversed norepinephrine and isoproterenol‐mediated inhibition of cell cycle re‐entry into the S‐phase but failed to inhibit the norepinephrine‐mediated hypertrophic response. Thus, at least in rat‐derived neonatal ventricular cardiomyocytes, sympathetic stimulation of the β_1_/_2_‐adrenergic receptor preferentially suppressed cell cycle re‐entry via recruitment of p38α MAPK.

A previous study from our lab reported that phorbol ester stimulation of NNVMs recruited p38α MAPK, as depicted by the increased phosphorylation state of the serine/threonine kinase, and the SB203580 inhibited kinase activity (Hertig et al., 2018). The treatment of NNVMs with the phorbol ester PDBu significantly reduced 5‐bromo‐2′‐deoxyuridine incorporation, and treatment with the p38α/β MAPK inhibitor SB203580 completely reversed PDBu‐mediated inhibition of cell cycle re‐entry into the S‐phase. The latter response further revealed that cell cycle re‐entry in response to PDBu/SB203580 treatment was observed in two distinct subpopulations of NNVMs, distinguished by the absence or de novo expression of the intermediate filament protein nestin, and a predominant response was identified in the latter subpopulation. By contrast, cell cycle re‐entry into the S‐phase following norepinephrine/SB203580 and isoproterenol/SB203580 treatment was selectively observed in the NNVM subpopulation that failed to induce nestin expression. Thus, despite p38α MAPK recruitment following PDBu and norepinephrine stimulation, the β_1/2_‐adrenergic receptor selectively inhibited the cell cycle re‐entry of the subpopulation of rat neonatal ventricular cardiomyocytes distinguished by the absence of de novo nestin expression.

The underlying mechanism attributed to the preferential cell cycle re‐entry of distinct subpopulations of NNVMs in response to β_1_/_2_‐adrenergic and phorbol ester stimulation in the absence or presence of SB203580 may be attributed in part to the recruitment of distinct protein kinase C isoforms. PDBu treatment of NNVMs for 24 h recruited the conventional isoform PKC‐α and novel isoforms PKC‐δ and PKC‐ε as depicted by protein downregulation (Kebbe et al., 2023). A previous study from our lab reported that pharmacological inhibition of conventional protein kinase C isoforms selectively suppressed PDBu/SB203580‐mediated de novo nestin expression (Kebbe et al., 2023). In parallel, pharmacological inhibition of novel PKC isoforms significantly attenuated PDBu/SB203580‐mediated cell cycle re‐entry of nestin^(−)^‐NNVMs (Kebbe et al., 2023). Thus, in response to phorbol ester treatment, conventional and novel PKC isoforms apparently target distinct NNVM subpopulations with regard to cell cycle re‐entry. To unequivocally reaffirm the pharmacological data, a siRNA approach was employed to selectively downregulate protein levels of the conventional isoform PKC‐α. First, a previous study reported that PKC‐α mediated the hypertrophic response of neonatal rat ventricular cardiomyocytes (Braz et al., 2002). Consistent with the latter paradigm, siRNA‐mediated PKC‐α protein downregulation significantly attenuated phorbol‐ester‐mediated increases in the surface area of NNVMs. Second, work from our lab reported that phorbol ester stimulation of neonatal rat ventricular fibroblasts inhibited re‐entry into the S‐phase of the cell cycle (Hertig et al., 2023). Data in the present study support a seminal role of PKC‐α as siRNA‐induced protein downregulation of the isoform abolished PDBu‐mediated inhibition of cell cycle re‐entry of neonatal rat ventricular fibroblasts. In parallel, siRNA‐induced PKC‐α protein downregulation failed to prevent PDBu‐mediated inhibition of 5‐bromo‐2′‐deoxyuridine incorporation in the nestin^(−)^‐NNVM subpopulation, reaffirming previous pharmacological data of a selective role of a novel PKC isoform in this particular subpopulation (Kebbe et al., 2023). Moreover, in PKC‐α‐depleted NNVMs, de novo nestin expression and subsequent cell cycle re‐entry was severely compromised following PDBu/SB203580 treatment. Thus, the siRNA approach revealed that PKC‐α in the presence of SB203580 selectively increased the appearance and facilitated the cell cycle re‐entry of the nestin^(+)^‐NNVM subpopulation (Kebbe et al., 2023). By contrast, norepinephrine‐mediated inhibition of cell cycle re‐entry of the nestin^(−)^_−_NNVM subpopulation was associated with the sustained preferential downregulation of the PKC‐ε isoform. Consistent with the latter observation, β‐adrenergic‐mediated apoptosis of adult rat ventricular cardiomyocytes occurred via a PKC‐ε‐dependent pathway (Shizukuda & Buttrick, 2001). Furthermore, the study by Rhode and colleagues revealed that treatment of neonatal rat ventricular cardiomyocytes with NE for 24 h preferentially downregulated PKC‐ε protein levels whereas PKC‐α expression remained unchanged (Rohde et al., 2000). Collectively, these findings indirectly support the premise that the novel PKC‐ε isoform may play a seminal role facilitating the cell cycle re‐entry of the nestin^(−)^‐NNVM subpopulation following stimulation with PDBu/SB203580 and norepinephrine/SB203580.

Previous studies have identified a panel of genes that were associated with cell cycle re‐entry and subsequent cytokinesis of ventricular cardiomyocytes (Cui et al., 2020; Kebbe et al., 2023; Liu et al., 2019) PDBu‐mediated attenuation of cell cycle re‐entry of NNVMs following a 24h treatment was associated with the mRNA upregulation of the cell cycle inhibitor CDKN2a (p16^INK4A^) and the transcriptional factor Runx1, reported to inhibit the cell cycle re‐entry of adult zebrafish ventricular cardiomyocytes (Kebbe et al., 2023; Koth et al., 2020; Mehdizadeh et al., 2021). Furthermore, PDBu treatment led to the mRNA downregulation of the guanine nucleotide exchange factor ECT2 required for cellular cytokinesis and the mitotic checkpoint kinase Bub1 implicated in the G_2_‐M phase (Kebbe et al., 2023; Kim & Gartner, 2021; Liu et al., 2019; Taubenberger et al., 2020; Wu et al., 2020). PDBu/SB203580 treatment of NNVMs for 24 h potentiated nestin mRNA levels, and the data was consistent with the de novo appearance of the intermediate filament protein in a subpopulation of NNVMs. By contrast, PDBu‐mediated mRNA upregulation of CDKN2a was unaffected by SB203580 treatment, whereas a partial significant attenuation of increased Runx1 mRNA levels was observed. Moreover, SB203580 treatment attenuated PDBu‐mediated Bub1 and ECT1 mRNA downregulation, but a significant difference was observed exclusively with Bub1. These findings were in part consistent with the data observed following a 72h treatment of NNVMs with PDBu and SB203580 and the pattern of gene expression and may have contributed in part to re‐entry into the S‐phase cell cycle of nestin^(−)^‐NNVMs and/or nestin^(+)^‐NNVMs (Kebbe et al., 2023). By contrast, NE treatment failed to upregulate CDKN2a and Runx1 mRNA levels, whereas ECT2 and Bub1 mRNA expression were significantly reduced. The NE‐mediated mRNA downregulation of Ect2 in NNVMs was consistent with a previous study revealing that sympathetic‐mediated stimulation of the β‐adrenergic receptor reduced Ect2 expression in mouse ventricular cardiomyocytes, translating to increased binucleation (Liu et al., 2019). However, SB203580 treatment failed to reverse NE‐mediated downregulation of ECT2 and Bub1 mRNA levels in neonatal rat ventricular cells, suggesting that p38α/β MAPK was not involved in the expression of these two genes in neonatal rat ventricular cardiomyocytes. Collectively, these data reaffirm the dichotomy of NE and PDBu signaling in NNVMs in the absence and presence of SB203580 and further support the premise that the pattern of mRNA expression associated with the re‐entry into the S‐phase of the cell cycle is apparently distinct between nestin^(−)^‐NNVMs and nestin^(+)^‐NNVMs.

**FIGURE 8:**
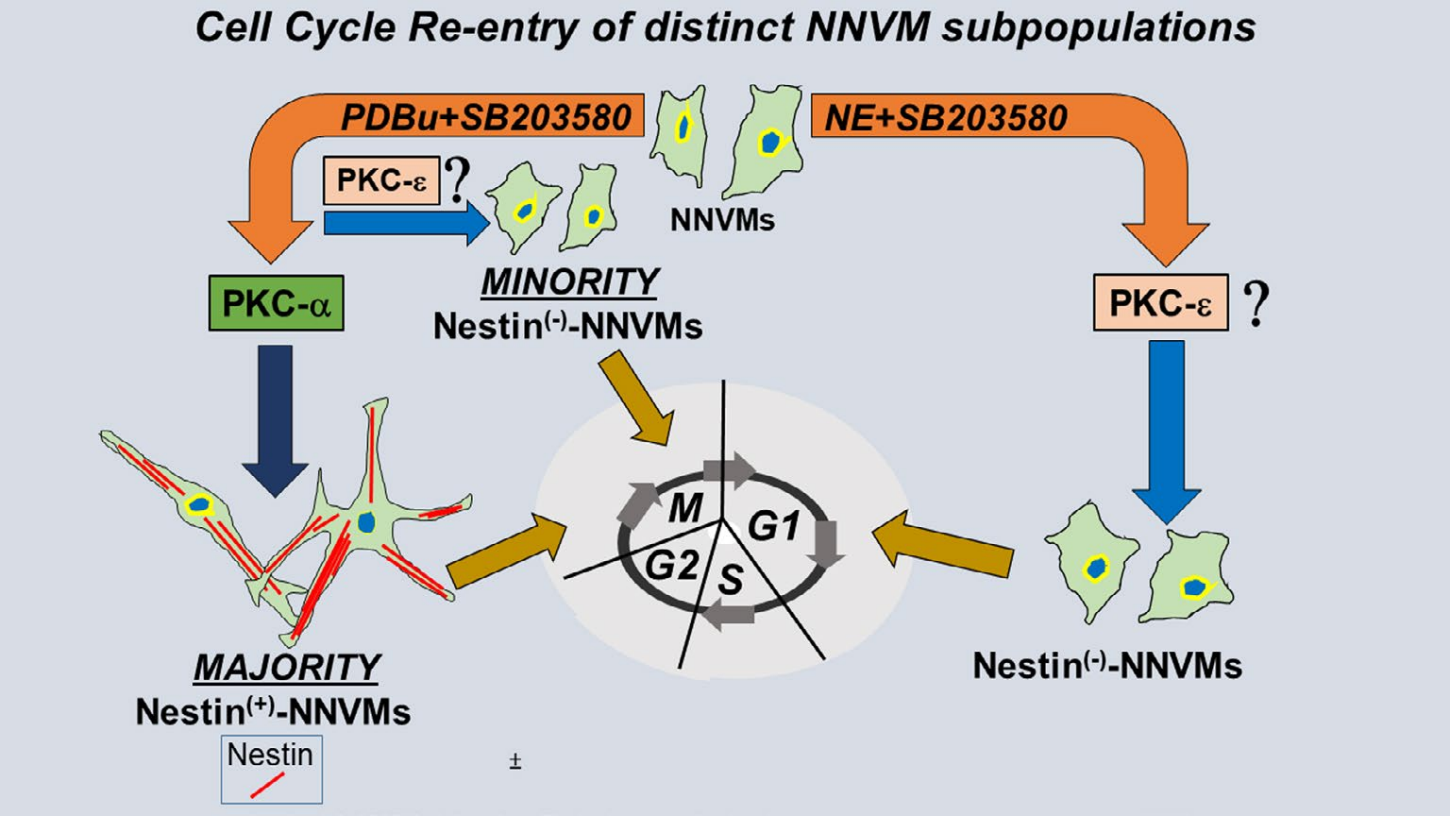
Cell cycle re‐entry of distinct subpopulations of NNVMs. The treatment of 1‐day old NNVMs with phorbol 12,13‐dibutyrate (PDBu) in the presence of the p38α/β MAPK inhibitor SB203580 led to the cell cycle re‐entry of two distinct subpopulations distinguished by the absence or de novo expression of the intermediate filament protein nestin. A siRNA approach targeting PKC‐α revealed that protein downregulation of the serine/threonine kinase selectively suppressed PDBu/SB203580‐mediated de novo nestin expression and cell cycle re‐entry of a subpopulation of NNVMs. By contrast, data from a previously published paper (Kebbe et al., 2023) and findings of the present study indirectly support the premise that the cell cycle re‐entry of the nestin^(−)^‐subpopulation in response to norepinephrine/SB203580 and PDBu/SB203580 treatment was driven by the novel isoform PKC‐ε. Additional experiments are warranted to unequivocally confirm the latter premise.

In conclusion, the present study has revealed that norepinephrine acting via the β_1/2_‐adrenergic receptor and PDBu preferentially facilitated re‐entry into the cell cycle of distinct subpopulations of NNVMs distinguished by the absence or de novo nestin expression in the presence of p38α/β MAPK inhibition, respectively. At the cellular level, activation of the conventional isoform PKC‐α concomitant with p38α/β MAPK inhibition selectively promoted the appearance and cell cycle re‐entry of the nestin^(+)^‐NNVM subpopulation (Figure 8). By contrast, previously published findings and data obtained in the present study indirectly support the premise that the novel PKC‐ε isoform may have driven, at least in part, the cell cycle re‐entry of the subpopulation of NNVMs distinguished by the absence of de novo nestin following concomitant PDBu/SB203580 or norepinephrine/SB203580 treatment (Kebbe et al., 2023) (Figure 8). Based on these data and the findings depicted in the study by Lui and colleagues (Liu et al., 2019), β‐blocker administration may have selectively prevented sympathetic‐mediated inhibition of cell cycle re‐entry of the subpopulation of mononucleated ventricular cardiomyocytes distinguished by the absence of de novo nestin expression following ischemic injury to the adult mammalian heart (Chen et al., 2023; Liu et al., 2019). However, the findings of the present study support the premise that pharmacological inhibition of p38α/β MAPK in the ischemically damaged adult mammalian heart may represent a more efficient approach to trigger the cell cycle re‐entry of both nestin^(−)^‐ and nestin^(+)^‐ventricular cardiomyocyte subpopulations (Arabacilar & Marber, 2015; Hertig et al., 2018, 2019; Kebbe et al., 2023; Li et al., 2005). Indeed, the latter response was observed following SB203580 administration to the ventricular apex‐resected 1‐day‐old neonatal rat heart, translating to a cardiac regenerative response (Hertig et al., 2019). Nonetheless, it remains presently speculative as to whether triggering the cell cycle re‐entry of one or both ventricular cardiomyocyte subpopulations is sufficient to promote at least a partial cardiac regenerative response in the ischemically damaged adult mammalian heart.

## AUTHOR CONTRIBUTIONS

Adrien Aubry: Immunofluorescence, Western blot, cell culturing, and QPCR. Mariana Kebbe: Immunofluorescence, Western blot, and cell culturing. Aya Al‐Katat: Western blot. Louis Villeneuve: Immunofluorescence. Angelino Calderone developed the study and wrote the initial draft of the paper.

## FUNDING INFORMATION

This work was funded by la Fondation de Recherche d'Institut de Cardiologie (FRIC; awarded to AC) and Canadian Institutes of Health Research (awarded to AC; grant PGT‐168859).

## CONFLICT OF INTEREST STATEMENT

All authors declare that there are no competing interests or conflicts.

## ETHICS STATEMENT

The use and care of laboratory rats was according to the Canadian Council for Animal Care and approved by the Animal Care Committee of the Montreal Heart Institute.

## CONSENT

All authors have approved the final version of the manuscript, are accountable for all aspects of the data, each author qualifies for authorship, and provide consent to publish.

## Supporting information


Figure S1.

## Data Availability

The datasets used and/or analyzed during the current study are available from the corresponding author on reasonable request.
