# Supplementary material for: Norepinephrine inhibits cell cycle re‐entry of neonatal rat ventricular cardiomyocytes characterized by the absence of de novo nestin expression
Source: Physiol Rep. 2025 Jul 30;13(15):e70488. doi: 10.14814/phy2.70488 (PMC12309976; doi:10.14814/phy2.70488)
Supplement: Supplementary file 1 — Figure S1. [file PHY2-13-e70488-s001.zip › Supplemental Figure Legends.docx]

**Figure S1 *Methods Flow Chart A***

Neonatal rat ventricular cardiomyocytes (NNVMs) were treated with norepinephrine (NE; 1 uM) in the absence or presence of the p38α/β MAPK inhibitor SB203580 (10 µM) for 24 hours to assess cell cycle re-entry into the S-phase via 5-bromo-2′-deoxyuridine (BrdU) incorporation and appearance of nestin^(+)^-NNVMs. In parallel, the expression pattern of genes associated with cell cycle re-entry and hypertrophy were determined following NE treatment of NNVMs in the absence or presence of SB203580 for a period of 24 hours.

**Figure S2 *Methods Flow Chart B***

Neonatal rat ventricular cardiomyocytes (NNVMs) were treated with phorbol 12,13-dibutyrate (PDBu; 100 nM) in the absence or presence of the p38α/β MAPK inhibitor SB203580 (10 µM) for 24 hours to assess cell cycle re-entry into the S-phase via 5-bromo-2′-deoxyuridine (BrdU) incorporation and appearance of nestin^(+)^-NNVMs. An siRNA approach targeting PKC-α was used to examine the role of the conventional protein kinase C isoform in the appearance and cell cycle re-entry of nestin^(+)^-NNVMs. In parallel, the expression pattern of genes associated with cell cycle re-entry and hypertrophy were determined following PDBu treatment of NNVMs in the absence or presence of SB203580 for a period of 24 hours.
