# Supplementary figures and images for: Norepinephrine inhibits cell cycle re‐entry of neonatal rat ventricular cardiomyocytes characterized by the absence of de novo nestin expression
Source: Physiol Rep. 2025 Jul 30;13(15):e70488. doi: 10.14814/phy2.70488 (PMC12309976; doi:10.14814/phy2.70488)

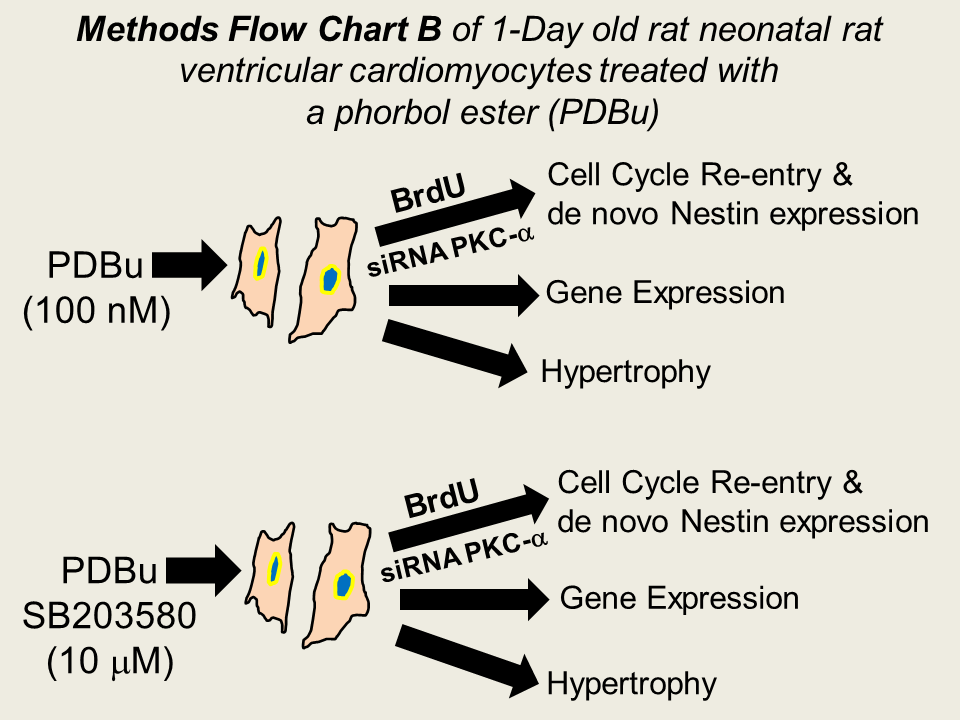

Supplement: Supplementary file 1 — Figure S1. [file PHY2-13-e70488-s001.zip › PHYSREP-2025-06-481-s03.tif]

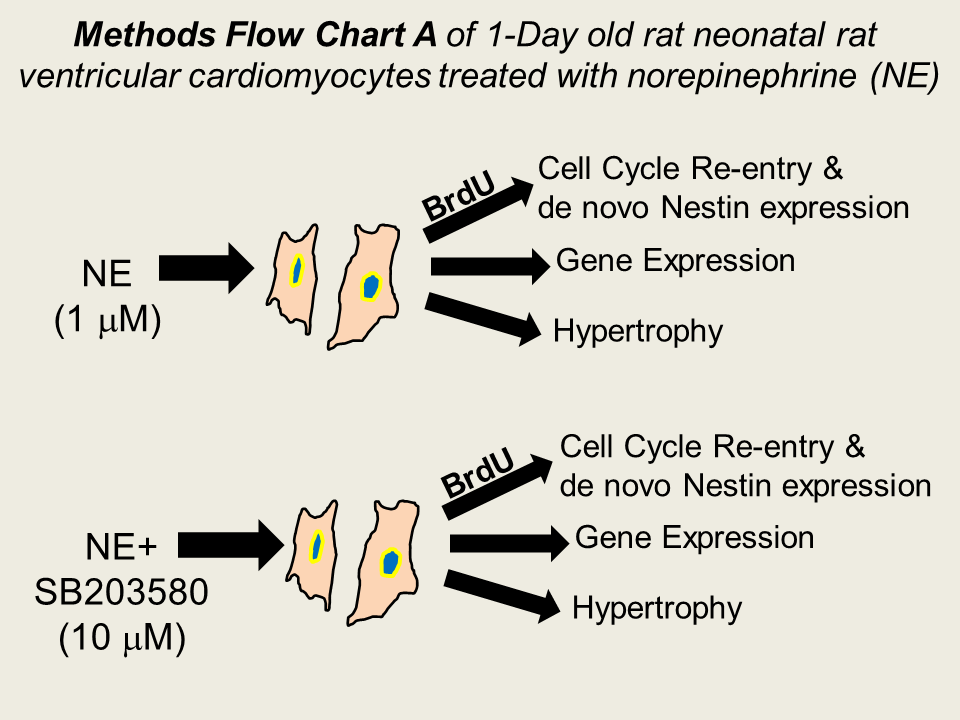

Supplement: Supplementary file 1 — Figure S1. [file PHY2-13-e70488-s001.zip › PHYSREP-2025-06-481-s02.tif]
